# Uncovering Analytical Patterns for Hazardous Components in Agricultural Production Systems

**DOI:** 10.3390/foods14183261

**Published:** 2025-09-19

**Authors:** Shiyu Deng, Xinxin Wu, Yongqiang Shi, Hany S. El-Mesery, Xinai Zhang

**Affiliations:** 1 School of Food and Biological Engineering, Jiangsu University, Zhenjiang 212013, China; 15387215710@163.com (S.D.); 19825549348@163.com (X.W.); shi_yongq@163.com (Y.S.); 2 School of Energy and Power Engineering, Jiangsu University, Zhenjiang 212013, China; elmesiry@ujs.edu.cn

**Keywords:** agricultural products, hazardous agents, pesticide residues, heavy metals, antibiotics, genetically modified organisms, detection technologies

## Abstract

Global food safety concerns underscore the critical importance of detecting hazardous components in agricultural production. This systematic review uncovers the prevalence and health impacts of common hazardous agents in agricultural commodities, including pesticide residues, heavy metals, mycotoxins, microbial contaminants, antibiotic residues, and genetically modified material. It thoroughly analyzes research progress in conventional detection methodologies. Furthermore, the review critically examines current challenges and future trajectories in analysis patterns, with particular emphasis on integrated technological approaches, field-deployable rapid detection devices, and the development of global standardized frameworks. This work aims to provide comprehensive technical guidance for the efficient and precise detection of hazardous components in agricultural products and to inform the advancement of robust food safety regulatory systems.

## 1. Introduction

Agricultural production, constituting the foundation of the human food chain, directly impacts public health and social stability [1,2,3]. Intensive agricultural practices and increasingly complex processing technologies have driven rising global detection rates of hazardous agents, including pesticide residues [4,5], heavy metal contamination [6], mycotoxins [7], microbial pollutants [8], antibiotic misuse [9], and genetically modified organisms (GMOs) [10,11]. Data from the World Health Organization (WHO) and the Food and Agriculture Organization of the United Nations (FAO) indicate that a significant proportion of global foodborne disease fatalities each year are directly caused by agricultural products containing hazardous substances that exceed regulatory standards [12,13]. These contaminants not only threaten human immunological, neurological, and reproductive systems through bioaccumulation but also pose chronic health risks such as carcinogenesis and systemic inflammation [14,15]. Therefore, developing efficient and precise analytical platforms has become imperative for ensuring food safety [16,17].

While demonstrating high accuracy as exemplified by techniques including high-performance liquid chromatography (HPLC) [18], gas chromatography-mass spectrometry (GC-MS), and enzyme-linked immunosorbent assay (ELISA), conventional approaches exhibit critical limitations: extended processing times, complex sample pretreatment protocols, and dependence on specialized laboratory infrastructure [19]. For instance, chromatographic methods typically require several hours to days for analytical completion, rendering them unsuitable for rapid on-site screening. Immunoassays, despite operational simplicity, suffer from cross-reactivity interference and inability to achieve multiplex detection [20]. Furthermore, these techniques show inadequate assay capabilities for trace-level contaminants, failing to meet regulatory monitoring requirements for hazardous agents in agricultural commodities. These limitations highlight the urgent need for next-generation analytical platforms [21].

Recent advances in biosensing platforms, nano-engineered interfaces, algorithm-assisted analytics, and CRISPR-based diagnostics have catalyzed innovation in agricultural product monitoring [22,23]. These developments include biosensors enabling in situ real-time surveillance of pesticide residues and heavy metal ions via bio-recognition-to-transduction coupling [24]; Artificial intelligence (AI)-driven spectral deconvolution models for rapid contaminant detection in complex matrices [24,25]; CRISPR-Cas analytical platforms providing genome-level resolution for GMO identification [26,27]. Critically, convergent technology integration exemplified by bio-sensor–nanomaterial–machine learning architectures [28,29], has emerged as a pivotal approach to transcend single-method constraints, delivering synergistic enhancements in assay velocity, sensitivity, and selectivity.

**Figure 1:**
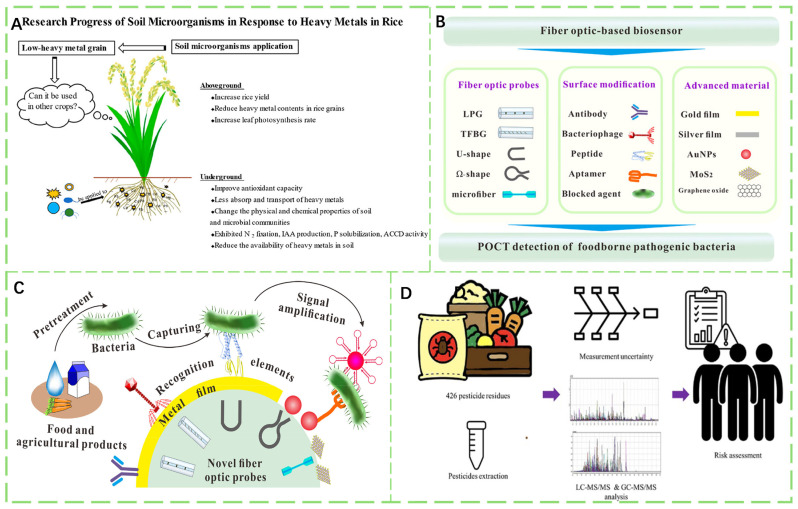
(**A**) Research progress of soil microorganisms in response to heavy metals in rice [30]. (**B**) POCT detection of foodborne pathogenic bacteria [31]. (**C**) Fiber-optic-based biosensor scheme for detecting foodborne bacteria [31]. (**D**) Methods for analyzing pesticide residues in various vegetables [32].

As shown in Figure 1A, soil heavy metal contamination leads to excessive heavy metal levels in crops, posing severe impacts on the ecological environment and the health of humans. With the outbreak of certain foodborne diseases, related detection technologies are also continuously developing (Figure 1B). Many emerging techniques for detecting foodborne bacteria continue to emerge (Figure 1C). As depicted in Figure 1D, for pesticide residue detection, methods such as liquid chromatography-tandem mass spectrometry (LC-MS/MS) and gas chromatography-tandem mass spectrometry (GC-MS/MS) are employed. There are numerous traditional detection methods, while novel approaches continue to emerge, such as the application of fluorescent probes in detecting pesticide residues in food. Rapid detection technologies based on electrochemistry are also advancing.

Despite significant advances in detection technologies [33], persistent challenges include imperfect standardization frameworks, inadequate portability of field-deployable instruments [34], and limited simultaneous multi-component detection capability, foodborne diseases impose enormous health and economic burdens. Future research priorities encompass developing cost-effective, high-throughput portable analytical platforms for rapid on-site screening in settings such as fields and markets; establishing globally unified monitoring standards and databases to facilitate cross-regional technology transfer; exploring intelligent biosensing systems leveraging synthetic biology for dynamic monitoring of emerging contaminants [35]. This review systematically examines hazard mechanisms of deleterious compounds, compares conventional and novel analytical techniques, and evaluates integrated methodological approaches. Our analysis aims to provide a theoretical foundation for optimizing agricultural product safety monitoring and to inform evidence-based food safety regulatory policies.

## 2. Types of Hazardous Components in Agricultural Products

### 2.1. Pesticide Residues

**Figure 2:**
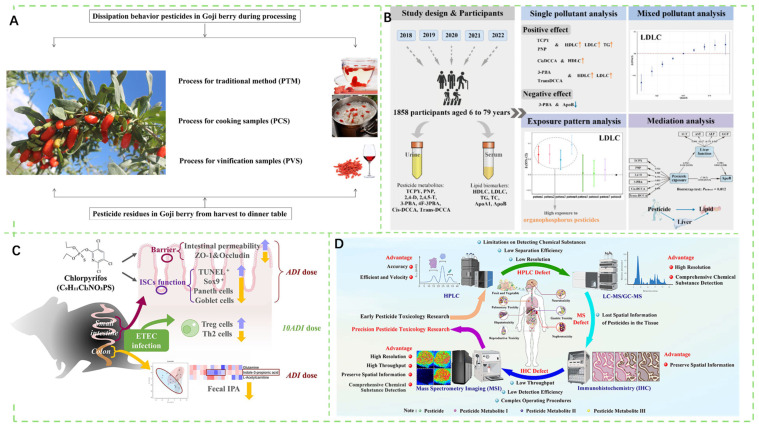
(**A**) Scheme showing Goji berry processing from harvest to dining table [43]. (**B**) Continuous low-level exposure to pesticides is inevitable in daily life [44]. (**C**) Chlorpyrifos damage the intestine [45]. (**D**) Techniques for pesticide toxicology research [46].

Pesticides constitute essential agricultural inputs for safeguarding crop yields globally. However, their ubiquitous use, exceeding million metric tons annually worldwide, raises significant concerns regarding residue accumulation [36,37]. While effectively protecting crop yields, improper application leads to persistent pesticide residues in agricultural products [38,39]. These pesticides enter ecosystems and the human body through multiple pathways, triggering a range of potential health risks, among which the gut dysbiosis and its cascading effects are of particular concern [40]. Improper use of pesticides leads to excessive residues in agricultural products, this pervasive contamination is of grave concern for global food security [41,42]. As shown in Figure 2A, excessive pesticide use jeopardizes human health, with residues persisting in food even after industrial processing. As confirmed, pesticide exposure was significantly associated with lipid metabolism (Figure 2B). For example, chlorpyrifos can inhibit intestinal stem cell proliferation and differentiation at the acceptable daily intake and disrupt immune responses at high doses (Figure 2C). Figure 2D displays that it is necessary to carry out in vivo pesticide toxicology research.

#### 2.1.1. Organophosphorus Pesticides

Although many classes of organic pesticides exist (e.g., carbamates, pyrethroids), organophosphorus pesticides were chosen as a representative group due to their wide application, well-characterized toxicological mechanisms, and significant health impacts. Other pesticide categories, such as neonicotinoids and herbicides, are discussed in the following sections. The toxicity mechanisms of organophosphorus pesticides are well-established [47]. These compounds act as irreversible inhibitors of *acetylcholinesterase* (AChE) [48], phosphorylating the serine residue within the enzyme’s active site [49,50]. This inhibition blocks acetylcholine (ACh) hydrolysis, leading to excessive ACh accumulation in synaptic clefts and consequent hyperstimulation of cholinergic nerves. Clinically, this manifests as muscarinic, nicotinic, and central nervous system effects [51].

The incidence of Parkinson’s disease significantly increases among agricultural workers exposed to organophosphorus pesticides over extended periods [52], with the mechanism being linked to mitochondrial dysfunction, abnormal aggregation of α-synuclein, and oxidative stress [53,54]. These findings underscore that pesticide residues pose not only acute health risks but also significant long-term neurodegenerative disease liabilities [55,56], and research by the University of Texas provides critical data on developmental vulnerability: their cohort study revealed that prenatal exposure to chlorpyrifos correlates with reduced working memory capacity and diminished prefrontal cortex gray matter volume in children, explicitly demonstrating the heightened susceptibility of the developing nervous system to pesticides compared to the adult system [57].

#### 2.1.2. Neonicotinoid Insecticides

Neonicotinoid insecticides, the most extensively deployed synthetic pesticides globally, exert deleterious effects extending far beyond agricultural contexts. These compounds induce irreversible activation of nicotinic acetylcholine receptors (nAChRs) in insect central nervous systems, triggering sustained neurotransmission that culminates in targeted organism paralysis and mortality [58]. Critically, their catastrophic neurotoxicity to pollinating insects and disruption of ecosystem equilibria represent the paramount concerns.

Chronic exposure to neonicotinoids at sublethal doses significantly reduces queen oviposition rate and impairs worker brood care behavior, directly threatening colony sustainability [59,60]. More critically, environmental persistence of these compounds initiates cross-kingdom biomagnification through pollen-nectar-aquatic insect-avian trophic transfer, triggering multi-trophic cascades [61]. Neonicotinoid seed treatments have attracted global attention. Large-scale field experiments evaluating winter oilseed rape crops treated with these compounds show variable effects on three bee species across Hungary, Germany, and the UK: negative impacts were observed in Hungary and the UK, while positive effects occurred in Germany. Notably, thiamethoxam-related negative effects in Hungary persisted through winter, leading to smaller spring colonies the following year. Additionally, reproduction in wild bees (Bombus terrestris and Osmia bicornis) correlated negatively with neonicotinoid residues, indicating that neonicotinoids reduce bees’ ability to establish new populations within one year of exposure [62,63]. These ecological disruptions underscore the critical disruption to the underlying ecological equilibrium [64,65].

#### 2.1.3. Herbicides

Herbicides such as glyphosate function by inhibiting *5-enolpyruvylshikimate-3-phosphate synthase* (EPSPS), thereby blocking aromatic amino acid synthesis in plants [66]. Their hazards now extend far beyond agricultural fields and have emerged as a focal point for public health and ecological security [67,68,69]. Concurrently, chronic exposure to glyphosate has been linked to elevated risks of certain cancers—an association that has fueled ongoing scientific debate and global regulatory scrutiny [70].

### 2.2. Heavy Metal Contamination

**Figure 3:**
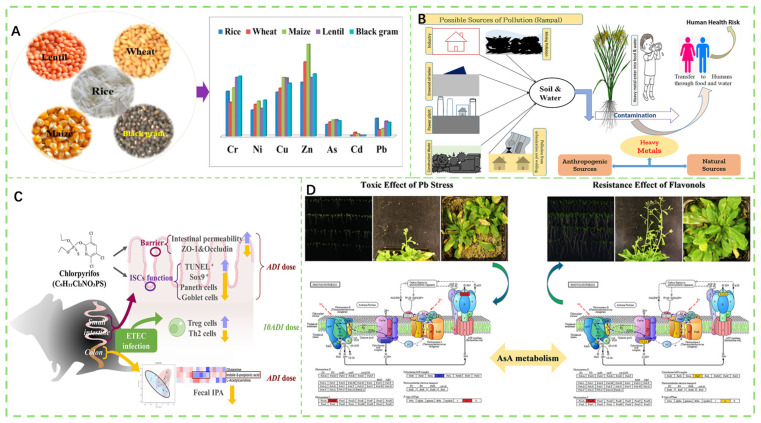
(**A**) Relative distribution of seven common heavy metals (Cr, Ni, Cu, Zn, As, Cd, Pb) in cereals and legumes [79]. (**B**) Sources of heavy metal pollution and its hazards to humans [80]. (**C**) Study on the mechanism of Cd accumulation in *Lactuca sativa* [81]. (**D**) The relationship between Pb stress and flavonols, as well as the functional mechanisms of flavonols [82].

Heavy metal contamination poses a critical threat to the safety of agricultural products [71,72,73]. These metals enter agroecosystems via mining, smelting, industrial effluents, and fertilizer application, and subsequently accumulate in the edible tissues of crops [74,75]. Once accumulated, their toxicity is governed by chemical speciation. Characterized by persistence, bioaccumulation potential, irreversible sequestration in soil–plant systems, and harm to human health, heavy metals are extremely difficult to remediate once introduced [76,77]. As shown in Figure 3A, growing cereals and legumes on contaminated soil may lead to metal transfer to the edible parts, potentially posing health risks to humans. Cities with more advanced industrialization exhibit more severe heavy metal pollution (Figure 3B). Cadmium contamination in agricultural products endangers the health of humans, and screening and breeding safe varieties with low cadmium accumulation (Cd-PSC) is an effective strategy to reduce cadmium contamination risks in dietary crops (Figure 3C). It has been confirmed that flavonols can enhance plant resistance to abiotic stress and also exhibit a remission effect under Pb stress (Figure 3D). The following subsections present seven common heavy metals (Cr, Ni, Cu, Zn, As, Cd, and Pb), each discussed separately [78].

#### 2.2.1. Cd

Cd contamination originates predominantly from phosphatic fertilizer application and irrigation with electroplating effluent [83,84]. Following bioaccumulation in rice (*Oryza sativa*) grains, Cd exposure induces Itai-itai disease, characterized by osteomalacia, osteoporosis, and progressive renal tubular dysfunction [85]. The epidemic-scale morbidity occurring in Toyama Prefecture, Japan, remains a seminal case documenting heavy metal-induced pathogenesis in human populations [86].

#### 2.2.2. Pb

Pb contamination primarily derives from atmospheric deposition of leaded gasoline particulates and leachate generated by discarded batteries. Pb exposure increases the risk of neurodevelopmental disorders in children [21,87]. Due to their activity patterns and the surrounding land use conditions, children have greater opportunities for lead exposure through soil contact [88,89]. In northern Taiwan, soil lead contamination and land use characteristics such as green spaces around residences are potentially associated with lead concentrations in children’s hair and nails, and also impact neurodevelopment [21]. For example, hair lead levels show a negative correlation with expressive language scores, while living near highways (a source of Pb exposure) may adversely affect children’s gross motor scores. Transplacental transfer further induces prenatal toxicity, manifesting as reduced neonatal weight and impaired cognitive development. In postindustrial regions, legacy contamination from historical leaded fuel emissions and unregulated battery disposal has resulted in persistent soil-water Pb exceedances, constituting an ongoing critical threat to pediatric health [90].

#### 2.2.3. As

Arsenic (As) contamination primarily originates from natural geological formations, groundwater leaching in gold mining districts, residual organoarsenical pesticides, and other agricultural and industrial activities [91]. Chronic arsenic exposure is causally linked to dermal hyperkeratosis, Bowen’s disease, and Blackfoot disease, an endemic peripheral vascular disorder induced by endothelial dysfunction. Critically, arsenic speciation analysis proves more clinically relevant than total concentration measurements: inorganic arsenite (As(III)) exhibits approximately 100-fold greater cytotoxicity than organic arsenobetaine [92]. The accumulation of inorganic As in rice (*Oryza sativa*) constitutes the primary exposure pathway for chronic arsenic poisoning across Asia [93,94]. Human consumption of As-contaminated water causes acute toxicity, while chronic exposure leads to effects ranging from skin lesions to cancer; recent studies have further linked arsenic exposure to intestinal diseases, type 2 diabetes, and various other cancers.

#### 2.2.4. Cr

Chromium is commonly introduced into agricultural soils through industrial effluents, sewage irrigation, and the application of certain fertilizers. Although trivalent chromium (Cr^3+^) is considered an essential trace element at very low levels, hexavalent chromium (Cr^6+^) is highly toxic and poses severe health concerns. Long-term exposure to Cr^6+^ can lead to oxidative stress, DNA damage, and increased cancer risk [95]. In cereals and legumes, chromium uptake is strongly influenced by soil pH and redox conditions, which affect its speciation and bioavailability. Monitoring chromium species is therefore critical for evaluating its actual health risk in agricultural products.

#### 2.2.5. Ni

Nickel contamination in grain crops primarily arises from industrial emissions, sewage sludge application, and phosphate fertilizers. Although nickel is involved in certain enzymatic processes in plants, excessive accumulation in food crops can be harmful to human health. Dietary exposure to elevated Ni levels has been associated with allergic reactions, respiratory disorders, and potential carcinogenicity. Cereals grown in contaminated soils often show higher nickel levels, raising concerns for populations relying heavily on these staples [96]. Strengthening surveillance of nickel residues in food products is thus necessary to prevent chronic exposure risks.

#### 2.2.6. Cu

Copper is an essential micronutrient required for normal physiological functions, including enzymatic activity and iron metabolism. However, excessive copper intake through food can result in gastrointestinal distress, oxidative stress, and liver damage. In agriculture, copper-based pesticides and fungicides are a major source of contamination in cereals and legumes. Continuous use of Cu-containing agrochemicals can lead to soil accumulation, which in turn elevates copper levels in edible crops [97]. Although moderate amounts are necessary for human health, careful regulation and monitoring of copper residues in food remain crucial to avoid toxicity.

#### 2.2.7. Zn

Zinc is another essential trace element that plays a vital role in growth, immune function, and cellular metabolism. However, high zinc concentrations in food crops may disrupt the balance of other trace metals and contribute to health problems such as nausea, vomiting, and impaired copper absorption [98]. Agricultural practices including excessive use of zinc-containing fertilizers and industrial pollution are major contributors to elevated Zn levels in cereals. While Zn deficiency is a global nutritional issue, excessive exposure from contaminated food sources should not be overlooked. Regular assessment of zinc content in staple grains is therefore important to ensure dietary safety.

### 2.3. Mycotoxin

**Figure 4:**
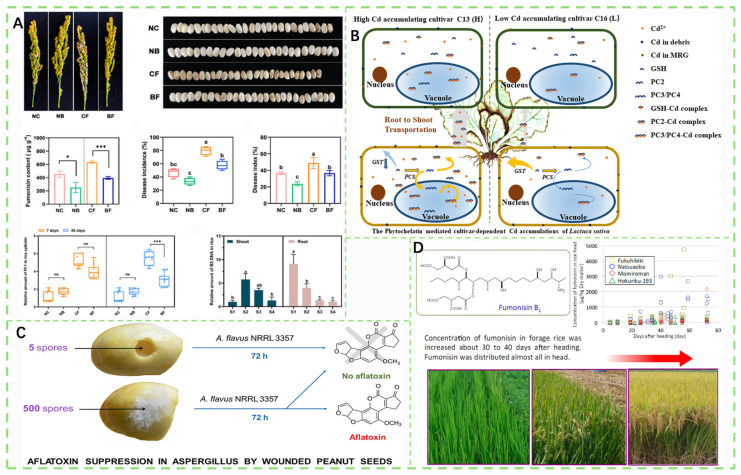
(**A**) Efficacy of endophytic fungal treatment in controlling rice spikelet rot disease and *fumonisin* accumulation in grains (*: *p* < 0.05, ***: *p* < 0.001, ns: no significant difference; lowercase letters (a–c) indicate significant differences among groups, with groups labeled different letters differing significantly at *p* < 0.05.) [102]. (**B**) Potential effects of peanut antitoxins on fungal development and aflatoxin formation during the interaction between peanuts and fungi [103]. (**C**) Results on the structure, processing fate, and origin pathways of *deoxynivalenol oligoglucosides* in cereal foods, along with the analytical methods employed [104]. (**D**) Feeding water *Oryza sativa* growth period *fumonisin* concentration changes [105].

Mycotoxins, toxic secondary metabolites produced by toxigenic fungi, contaminate globally distributed agricultural commodities including cereals, animal feeds, nuts, and dairy products [7,99]. They affect a significant portion of annual crop production worldwide, causing substantial economic losses and posing severe health threats to humans and livestock due to their potency even at trace exposure levels [100]. Figure 4A illustrates *Fusarium* head blight (FHB), a devastating fungal disease of wheat (*Triticum aestivum*). The proliferation of *Fusarium* leads to rice spike rot (RSRD), which severely reduces crop yields, produces mycotoxins, and poses a threat to human health [101]. Common soil fungi like *Aspergillus flavus* and parasitic Aspergillus serve as opportunistic pathogens invading peanut seeds before harvest. These fungi frequently generate carcinogenic aflatoxins that endanger human and animal health through the food chain (Figure 4B). Deoxynojingia erinacei, a common grain contaminant, contains conjugated masking forms such as deoxynojingia erinacei-3-glucoside in infected crops. The presence of these hidden mycotoxins in human diets has become a concerning public health issue (Figure 4C). Mycotoxin contamination remains a global concern, with *Fusarium* species being primary toxin-producing fungi in temperate regions. Oryzanol, detected at high frequencies in crops, disrupts sphingolipid metabolic pathways, causing multiple health hazards to both humans and livestock (Figure 4D).

#### 2.3.1. Aflatoxin B_1_

Aflatoxin B_1_ (AFB_1_), one of the most hazardous mycotoxins [106,107], is metabolically activated by hepatic CYP3A4 to AFB_1_-8,9-epoxide, forming DNA adducts that drive tumor suppressor gene mutations [108,109]. Classified as a Group 1 carcinogen by the International Agency for Research on Cancer (IARC), *AFB*_1_ exhibits acute toxicity, carcinogenicity [110], and teratogenicity—ranking among the most potent naturally occurring carcinogens [106,110]. In subtropical regions, contamination of *Arachis hypogaea* (peanuts) and *Zea mays* (maize) significantly elevates human cancer risk upon dietary exposure [111].

#### 2.3.2. Deoxynivalenol

Deoxynivalenol (DON) binds the ribosomal 60S subunit, activating mitogen-activated protein kinase (MAPK) pathways that trigger apoptosis in intestinal epithelial cells [112]. Specifically, this trichothecene mycotoxin causes acute enterotoxicity manifesting as emesis and diarrhea, while chronic exposure impairs immune function [113,114]. During storage of *Triticum aestivum* (wheat) and *Zea mays* under warm temperatures and high relative humidity, toxigenic *Fusarium graminearum* proliferates, generating DON that compromises grain quality and safety [115,116].

#### 2.3.3. Fumonisin B_1_

*Fumonisin B*_1_ (FB_1_) inhibits ceramide synthase, disrupting sphingolipid metabolism and compromising membrane integrity [117,118]. Linked to hepatorenal toxicity and esophageal carcinogenesis, FB_1_ concentrations in *Zea mays* from high-incidence esophageal cancer regions are significantly higher than those in low-risk areas [119,120], providing etiological evidence and highlighting the imperative for agricultural control [121,122].

### 2.4. Microbial Contamination

**Figure 5:**
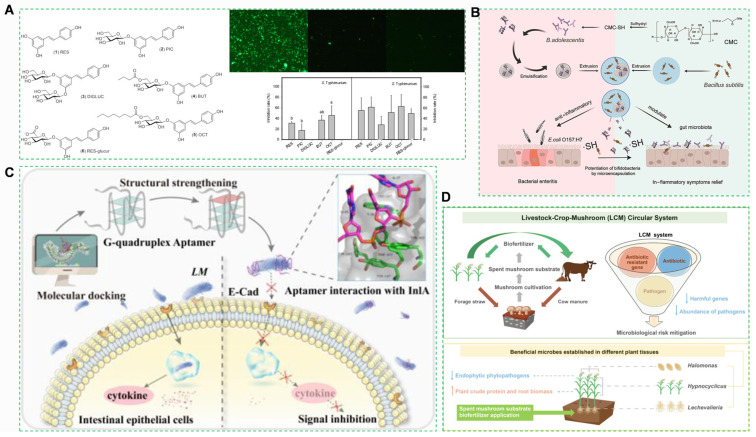
(**A**) Resveratrol and its derivatives have efficacy in inhibiting *Salmonella typhimurium* and *Escherichia coli* O157:H7 (Different lowercase letters (a, b, ab) above the bars indicate significant differences among groups (*p* < 0.05), and mixed letters represent an intermediate level.) [131]. (**B**) Microcapsules can alleviate inflammation in mice with bacterial enteritis induced by *Escherichia coli* O157:H7 [132]. (**C**) The Livestock–Crop–Mushroom (LCM) circular production model [133]. (**D**) A G4 structure aptamer specific to *InlA* with high thermal and chemical stability was designed with the aid of in silico techniques [134].

Foodborne pathogens cause a large number of annual illnesses globally, with agricultural products implicated in a high proportion of cases [123]. Critical risk determinants include: minimal infective doses, as exposure to a small number of viable cells of enterohemorrhagic *Escherichia coli* O157:H7 can initiate infection [114,124,125]; extended environmental persistence, as *Salmonella enterica* maintains viability for long periods on the phyllospheres of leafy vegetables; and cold-chain amplification, as *Listeria monocytogenes* multiplies at refrigeration temperatures in chilled foods [126]. *Salmonellosis* has become a serious public health problem due to the spread of antibiotic-resistant strains, and the treatment of O157:H7 infections is limited. Therefore, there is a need to investigate alternative or adjunctive antibiotic therapies [127]. Inhibiting pathogen adhesion to intestinal epithelium can prevent infections, and some probiotics, food-borne bacteriostatic agents, and anti-adhesive inhibitors have shown relevant potential (Figure 5A). Prolonged or extensive use of antibiotics can increase drug resistance, reduce the efficacy of antibiotics, accumulate in the body, and cause disturbances in the gastrointestinal flora as well as various diseases [128]. For instance, *Escherichia coli* O157:H7 can induce intestinal inflammation (Figure 5B) and microcapsules can alleviate such inflammation in mice with bacterial enteritis induced by this pathogen. *Listeria monocytogenes*, a common foodborne pathogen, can infect immunocompromised individuals and pregnant women, causing severe diseases such as sepsis and meningitis [129]; thus, exploring inhibitory approaches to control *L. monocytogenes* infection is of great significance (Figure 5C). Fecal-derived biofertilizers pose risks and challenges. Co-composting technology represents a feasible approach for converting such wastes (Figure 5D). Fecal-derived biofertilizers pose risks due to pathogenic microorganisms, as feces contain various pathogens whose application to soil may stimulate the proliferation of human pathogenic bacteria such as *Escherichia coli* and *Listeria* [130].

#### 2.4.1. *Escherichia coli*

*Escherichia coli* O157:H7 expressing Shiga toxin *Stx2* binds globotriaosylceramide (Gb3) receptors on renal glomerular endothelia, triggering BAX/BAK-mediated mitochondrial apoptosis and subsequent hemolytic uremic syndrome (HUS) [135]. Separately, FB_1_, linked to hepatorenal toxicity and esophageal carcinogenesis, exhibits significantly higher concentrations in *Zea mays* from high-incidence esophageal cancer regions compared to low-risk areas [136,137]. This provides etiological evidence and highlights the need for agricultural control measures.

#### 2.4.2. *Salmonella*

*Salmonella enterica* serovar Kiambu utilizes CsgD-regulated curli fimbriae to form persistent biofilms, evading post-harvest sanitization [138]. In EU melon contamination events, internal colonization via rind micro-wounds occurs at substantial levels, reducing the infectious dose compared to surface contamination [139]. This ecological adaptation undermines decontamination efficacy across supply chains [140,141].

#### 2.4.3. *Listeria monocytogenes*

*Listeria monocytogenes* employs *InlA*-mediated intestinal invasion and *listeriolysin O* (LLO)-driven phagosomal escape to cross placental barriers (with a high vertical transmission rate) [142]. A Danish cheese outbreak involving hypervirulent ST6 (CC1) increased miscarriage risk among pregnant women, correlating with enhanced E-cadherin binding affinity from an *inlA* F236Y mutation [143]. Its psychrotrophic proliferation at refrigeration temperatures establishes *L. monocytogenes* as a critical threat to immunocompromised populations via refrigerated ready-to-eat foods [144,145].

### 2.5. Antibiotic Residues

**Figure 6:**
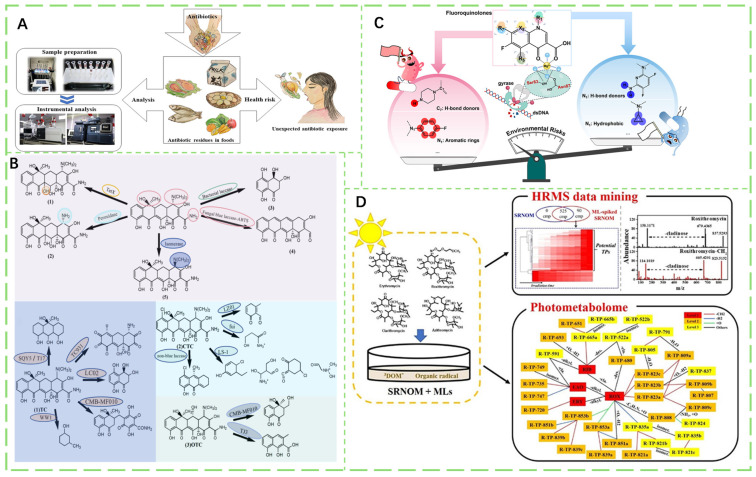
(**A**) Antibiotic residues in food, extraction, analysis, and health issues related to humans [148]. (**B**) Some pathways for microbial degradation of tetracycline antibiotics [154]. (**C**) The role of fluoroquinolone production intermediates in promoting environmental antibiotic resistance [155]. (**D**) The natural fate of macrolide antibiotics in aquatic environments [156].

Since their advent in the 1930s, antibiotics have initially been primarily used for treating and preventing diseases in humans and animals. In 1950, an additional value was discovered: they can promote growth in food-producing animals and improve feed utilization efficiency, leading to their widespread use as feed additives [146]. A non-negligible consequence of excessive antibiotic use in these animals is residual accumulation in edible tissues. Residual antibiotics in food may directly cause diseases through low-dose exposure and indirectly harm humans by adversely affecting antibiotic resistance (Figure 6A). The widespread misuse of veterinary antibiotics results in unmetabolized drugs entering agroecosystems via organic fertilizers, establishing a “soil-crop-human” translocation pathway with global implications [147]. Core risks encompass chronic low-dose exposure-driven antimicrobial resistance (AMR) pandemics and ecological destabilization [148,149]. For example, tetracyclines (TCs), a class of broad-spectrum antibiotics widely used in animal husbandry and medicine [150], have persisted and spread in the environment due to overuse and improper disposal [151]. These residues may accumulate in humans through the food chain, posing health risks at certain concentrations. Innovative approaches are needed to mitigate their environmental persistence and associated risks (Figure 6B). Fluoroquinolones, another class of broad-spectrum antibiotics with potent bactericidal properties, are widely used in clinical and veterinary settings. Excreted in unmetabolized forms, they have become widespread in sediments, soils, and aquatic environments, sharply increasing antibiotic resistance (Figure 6C). Macrolides (MLs) are important antibiotics for human therapy, listed by the World Health Organization (WHO) as critically important antimicrobials of the highest priority in human medicine, and are effective in treating respiratory tract and genital infections [152,153]. Macrolide antibiotics are widely present in aquatic environments, and their role in the persistent spread of antimicrobial resistance has raised concerns among scientists and the public regarding their fate in wastewater and surface water (Figure 6D).

#### 2.5.1. Tetracycline Antibiotics

Tetracyclines persist in soils with over 40% of residues forming stable complexes with humic substances [157]. These residues deposit in bone tissue, disrupting calcium metabolism to cause premature epiphyseal closure in children, while enhancing *tet(M)* plasmid conjugative transfer that disseminates multidrug resistance. In intensive livestock regions, tetracycline accumulates in crops, threatening human health [158].

#### 2.5.2. Fluoroquinolone Antibiotics

Fluoroquinolone antibiotics, characterized by high hydrophilicity, act by inhibiting bacterial DNA gyrase. However, their clinical use may significantly increase the risk of tendon rupture and induce carbapenem resistance mediated by the *bla_nam_*_−1_ gene [159,160].

#### 2.5.3. Macrolide Antibiotics

Macrolide antibiotics exhibit strong binding capacity to humus and disrupt the gut microbiota, conferring obesogenic and diabetogenic risks. Additionally, expression of the *ermB* gene reduces the clinical efficacy of erythromycin. Such dysbiosis impairs host immunity and diminishes therapeutic outcomes [161].

To provide a concise comparison, the hazardous components in agricultural products and their major detection methods, including both traditional and emerging techniques, are summarized in Table 1.

## 3. Traditional Detection Methods

### 3.1. Chromatographic Analysis

**Figure 7:**
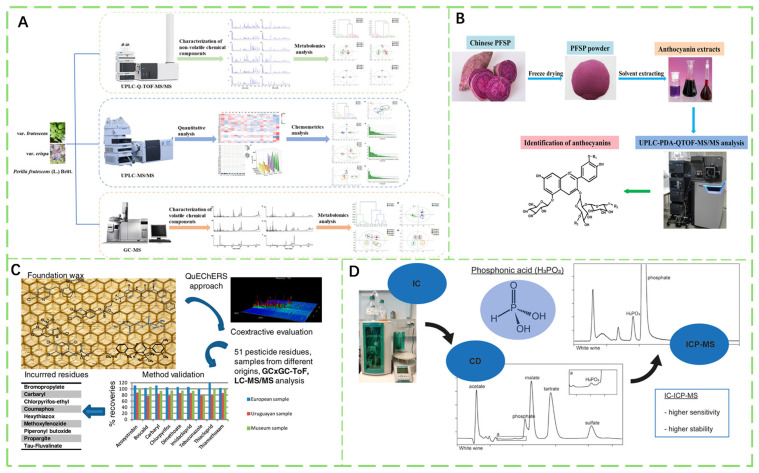
(**A**) Three methods were employed for qualitative analysis and content determination of fluticasone propionate in different medicinal plant parts [187]. (**B**) Identification and Quantification of Anthocyanins in Sweet Potatoes Using UPLC-PDA and UPLC-QTOF-MS/MS [188]. (**C**) Conventional analysis of pesticide residues in beeswax [189]. (**D**) Ion chromatography conductivity detection or ion chromatography inductively coupled plasma mass spectrometry for the determination of polar pesticides including phosphonates [190].

Chromatographic techniques remain the definitive confirmatory method for hazardous compounds in agricultural products, leveraging high separation efficiency and multiresidue screening capabilities [167,168]. Innovations in chromatographic column hardware and hyphenated systems have significantly improved detection accuracy while substantially reducing analysis time [169]. For example, high-performance liquid chromatography (HPLC) enables quantitative detection of aflatoxins and carbamate pesticides within regulatory limits of these substances through differential partitioning between a C_18_ stationary phase and a methanol/water mobile phase [170,171,172]. The combination of various chromatographic techniques has made the detection of components and related pesticide residues in crops more convenient and efficient (Figure 7A).

#### 3.1.1. Ultra-Performance Liquid Chromatography

Ultra-performance liquid chromatography (UPLC), which evolved from conventional HPLC [143], employs sub-2-μm particles to achieve significantly higher column efficiency and much faster analysis speeds [173,174]. The reduced particle size minimizes mass transfer resistance, enhancing separation of structural isomers. Coupled with high-resolution mass spectrometry, it enables untargeted screening of novel contaminants, revolutionizing contaminant discovery in agricultural products [175]. Simultaneously, the combination of UPLC and mass spectrometry can also identify and quantify the beneficial components in crops that are advantageous to the human body (Figure 7B).

#### 3.1.2. Comprehensive Two-Dimensional Gas Chromatography

Comprehensive two-dimensional gas chromatography (GC × GC) employs orthogonal separation mechanisms: a non-polar first-dimension column (boiling point-based separation) coupled to a polar second-dimension column (polarity-based separation) [176,177]. This configuration increases peak capacity by more than 10-fold and enhances resolution of co-eluting interferents in lipid-rich matrices, and can be used for detecting pesticide residues (Figure 7C). GC × GC achieves detection of persistent contaminants compliant with regulatory standards, thus establishing its critical role in environmental and food safety monitoring [169,178,179].

#### 3.1.3. Ion Chromatography

Ion chromatography (IC) stands as the exclusive mainstream technique for simultaneous quantification of glyphosate and its primary metabolite aminomethyl phosphonic acid (AMPA) at ultra-trace levels [179,180,181]. It uniquely resolves highly polar, ionic contaminants through suppressed conductivity detection (Figure 7D). When coupled with inductively coupled plasma mass spectrometry, this platform enables precise arsenic speciation analysis, distinguishing toxic inorganic arsenite from the less hazardous organic arsenobetaine (AsB) with high accuracy [182,183,184]. This capability is critical for refining heavy metal risk assessments in staple crops (e.g., quantification of inorganic arsenic in *Oryza sativa*) and elucidating speciation-dependent toxicity profiles [185,186].

### 3.2. Spectroscopic Analysis

Spectroscopic techniques characterize molecular interactions with electromagnetic radiation through precise measurement of absorption, emission, or scattering phenomena at defined wavelengths, enabling both qualitative screening and quantitative determination of analytes in complex agricultural matrices [191,192]. Infrared spectroscopy is commonly used for the identification of molecular functional groups, while Raman spectroscopy is primarily employed for the characterization of backbone structures [193]. Some vibrations can be measured by both techniques, although the intensities of these vibrations differ significantly [194,195]. A drawback of Raman spectroscopy is its inherently weak signal and susceptibility to fluorescence interference [196,197,198]. However, compared to infrared spectroscopy, water generally does not interfere with Raman analysis of samples—an advantage that enables Raman spectroscopy to be applied in on-site detection scenarios including agricultural and environmental samples [199,200].

#### 3.2.1. Laser-Induced Breakdown Spectroscopy

**Figure 8:**
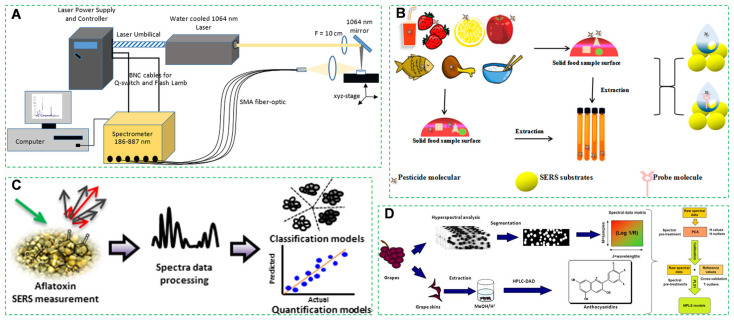
(**A**) LIBS experimental setup [221]. (**B**) Surface-enhanced Raman spectroscopy for pesticide residue detection [222]. (**C**) Surface-enhanced Raman spectroscopy for detecting *Aspergillus flavus* toxins in corn [223]. (**D**) Near-infrared hyperspectral imaging technology for anthocyanin screening in Vitis vinifera [224].

Laser-induced breakdown spectroscopy (LIBS) utilizes high-energy pulsed lasers to generate transient plasma on sample surfaces [201], enabling in situ quantification of heavy metals based on atomic emission spectroscopy and has been employed for qualitative and quantitative measurements of elemental composition across various matrices, including solids, liquids, and gases (Figure 8A) [202]. This technique achieves sample preparation-free analysis with rapid measurement cycles and high detection sensitivity, providing real-time soil contaminant monitoring critical for precision agriculture [203,204].

#### 3.2.2. Surface-Enhanced Raman Spectroscopy

Surface-enhanced Raman spectroscopy (SERS) utilizing gold nanostar substrates achieves significant signal enhancement of aflatoxin B_1_ molecules, enabled by the substrates’ surface plasmon resonance effect [205,206]. This ultrasensitive technique is therefore highly suitable for trace-level detection of fungal toxins [77,207,208]. Its exceptional sensitivity and specificity position SERS as a promising platform for food safety monitoring [209,210]. For instance, it enables rapid and accurate identification of low-concentration aflatoxins in nut-derived agricultural products (Figure 8B) [211,212]. Meanwhile, it exhibits excellent sensitivity for the detection of a broad range of pesticides as well as single-molecule pesticides, facilitating its adoption as an alternative detection technique for rapid pesticide analysis [213,214]. An increasing number of studies have utilized SERS for the rapid detection of pesticide residues in food products (Figure 8C).

#### 3.2.3. Near-Infrared Hyperspectral Imaging

Hyperspectral imaging is an emerging and rapidly developing non-destructive food analysis technique [215], typically performed in the visible-shortwave near-infrared or near-infrared spectral regions [216]. In recent years, the application of hyperspectral analysis in the food sector has increased significantly (Figure 8D). Near-infrared hyperspectral imaging (NIR-HSI) acquires concurrent spectral and spatial data from agricultural products [217,218]. Integrated with 3D convolutional neural networks (3D-CNNs), this technology enables non-destructive detection of internal quality attributes in Malus domestica (apple), including defect identification and biochemical quantification [219,220].

### 3.3. Electrochemical Methods

Electrochemical methods are increasingly used for detecting contaminants such as heavy metals, pesticide residues, and antibiotics in agricultural products. These techniques convert chemical interactions into measurable electrical signals (current, potential, or impedance), enabling rapid and sensitive detection. Their advantages include low cost, simple operation, fast response, and potential for on-site application. For instance, modified electrodes have been employed to monitor Cd and Pb in cereals, while enzyme-based biosensors have been applied to detect organophosphate pesticides. Nevertheless, electrochemical approaches are often affected by matrix interference and limited stability, which restrict large-scale use. Recent advances in nanomaterials and portable sensor design are helping to overcome these drawbacks, making electrochemical methods a promising complement to chromatographic and spectroscopic analysis [225].

### 3.4. Immunoassays

**Figure 9:**
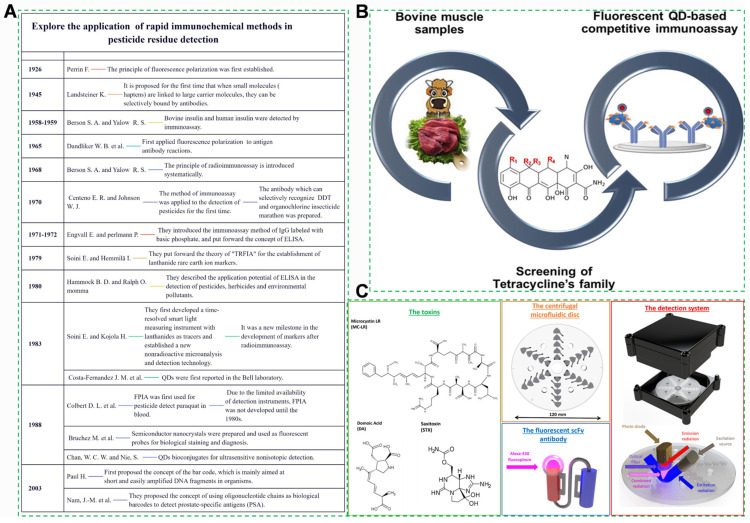
(**A**) Application of immunochemical methods in pesticide residue detection based on biotechnology [241,242,243,244,245,246,247,248,249,250,251,252] (**B**) Quantum dot (QD) fluorescence immunoassay for the detection of tetracycline antibiotics in bovine muscle [253] (**C**) Novel microfluidic analytical sensing platform for the simultaneous detection of three algal toxins in water [254].

Immunoassays are analytical techniques based on the specific and reversible binding between antigens (target harmful components) and antibodies [226]. Antigens in agricultural products are typically small molecules, such as pesticides, mycotoxins [227], veterinary drugs, or heavy metal complexes like chelated heavy metals, which can induce an immune response in animals to produce specific antibodies. Immunoassays, with a mature development history in pesticide residue detection, leverage this principle (Figure 9A). In the detection process, the interaction between the target antigen and its corresponding antibody forms an antigen–antibody complex, which is then quantified through signal amplification systems [228]. Such high specificity enables immunoassays to selectively identify trace harmful components in complex agricultural matrices without extensive sample pretreatment. Immunoassays represent a central approach for on-site rapid testing of agricultural products, leveraging their high specificity and operational simplicity [229].

#### 3.4.1. Quantum Dot-Labeled Immunochromatography

Quantum dots, a type of semiconductor nanocrystal, emit high-intensity fluorescence upon excitation, offering substantially higher fluorescence signal intensity than colloidal gold labels. This enables ultratrace target capture and marked improvement in sensitivity [230]. The technology has achieved significant breakthroughs in agricultural product safety testing [231].

For ultrasensitive mycotoxin detection, such as aflatoxin B_1_ in corn, this technology achieves an extremely low detection limit, representing a substantial improvement over traditional colloidal gold assays [232]. The technology can also further detect pesticide residues and antibiotic residues in agricultural products and food (Figure 9B) [233]. It significantly enhances the efficiency and accuracy of agricultural product safety screening, serving as a critical safeguard against the entry of non-compliant products into the market [234].

#### 3.4.2. Recombinant Antibody Technology

Recombinant antibody technology leverages phage display libraries to isolate high-affinity single-chain antibodies (scFv) and utilizes large-scale Escherichia coli production to achieve low batch-to-batch variation [235]. For example, recombinant scFv antibodies demonstrate substantially higher affinity for clenbuterol compared to conventional antibodies, achieving a detection limit in pork samples well below the national standard, and reducing false positive rates markedly [236]. Such advancements significantly improve antibody quality and stability, lower detection costs, and establish a robust foundation for the broad application of immunoassays in agricultural product safety testing [237].

#### 3.4.3. Enzyme-Linked Immunosorbent Assay (ELISA)

ELISA utilizes enzyme-labeled antibodies or antigens, where the enzyme activity measured via colorimetric signals is correlated with the concentration of the target analyte [238]. ELISA is widely applied due to its high sensitivity, low cost, and suitability for high-throughput screening [239]. The combination of ELISA technology and recombinant antibody technology can also be utilized for the detection of relevant toxins (Figure 9C). The integrated, miniaturized design of microfluidic ELISA significantly enhances assay efficiency, reduces costs, and enables field-deployable rapid screening for large-scale agricultural sample analysis [110,240]. To facilitate a clearer comparison, the advantages and disadvantages of major traditional analytical methods are summarized in Table 2.

**Table 2:** Comparison of Traditional Detection Methods.

Technique Category	Typical Methods	Advantages	Disadvantages	SERS
Chromatography	HPLC, UPLC, GC × GC, IC	High separation efficiency; accurate qualitative and quantitative analysis; strong capability for multi-residue detection	Complex sample pretreatment; long detection cycle; expensive instrumentation; unsuitable for on-site detection	[255]
Spectroscopy	IR, Raman, LIBS, SERS, NIR-HSI	Non-destructive; rapid detection; potential for online/field deployment	Limited sensitivity; strong matrix interference; fluorescence interference in some techniques	[256]
Immunoassays	ELISA, colloidal gold test strips, quantum dot immunochromatography	High specificity; easy to operate; suitable for large-scale rapid screening	Susceptible to cross-reactivity; limited sensitivity; antibody preparation is costly	[257]

### 3.5. Summary of Strengths and Limitations of Traditional Methods

Traditional analytical methods—including chromatographic, spectroscopic, electrochemical, and immunological approaches—remain the cornerstone of food contaminant detection. Each technique has clear advantages but also faces notable drawbacks. Chromatographic analysis provides high precision and established reliability, yet it requires complex pretreatment, long analysis time, and costly equipment. Spectroscopic techniques are rapid and non-destructive, but their sensitivity may be inadequate and results can be influenced by food matrices. Electrochemical methods are low-cost and portable, allowing for fast response, though they are often affected by background interference and stability issues. Immunoassays offer high specificity and are suitable for large-scale screening, but problems such as cross-reactivity, limited durability, and difficulty in multiplexing still exist.

Taken together, these shortcomings restrict the large-scale deployment of traditional approaches. They also explain the growing research interest in emerging technologies, which aim to overcome such limitations and provide more practical solutions for routine food safety monitoring.

## 4. Emerging Detection Technologies

### 4.1. Biosensor Technology

**Figure 10:**
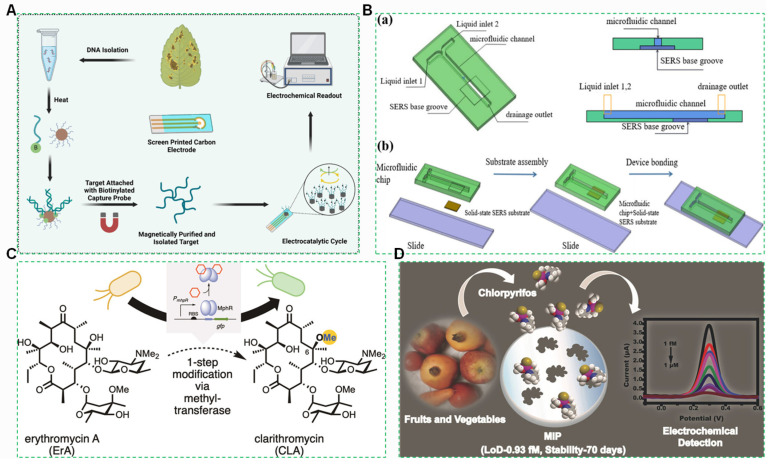
(**A**) rapid electrochemical biosensor diagnostic for *botrytis* ssp. causing botrytis gray mold of temperate legumes [274]. (**B**) 3D porous silicon carbide SERS microfluidic chip for pesticide residue detection: (**a**) Microfluidic chip design diagram; (**b**) Schematic diagram of microfluidic chip and SERS substrate assembly [275]. (**C**) Schematic diagram of *MphR*-mediated one-step modification of erythromycin A to clarithromycin and biosensing mechanism [276]. (**D**) Schematic diagram of MIP-based electrochemical detection of chlorpyrifos in fruits and vegetables and its agricultural application [277].

Biosensors are analytical devices that integrate biorecognition elements with transducers to convert specific biological interactions with target harmful components into measurable physical or chemical signals [258]. The biorecognition element selectively binds to analytes such as pesticides, mycotoxins, veterinary drugs, heavy metals, or microbial toxins in agricultural matrices (Figure 10A), inducing a biological response. The transducer then translates this response into a quantifiable signal, enabling sensitive and real-time detection without extensive sample pretreatment [259].

#### 4.1.1. Fully Integrated Microfluidic Biochips

Fully integrated microfluidic biochips represent a highly miniaturized detection platform [151,260]. This technology incorporates: an ultrasonic extraction module, enabling in situ solid–liquid separation and precise pH adjustment for fruit or vegetable purées; Magnetic nanoparticles for targeted capture of organophosphorus pesticides; An integrated detection core combining gold nanocone electrodes with quantum-dot-encoded microsphere systems [261]. This architecture establishes a complete “sample-to-answer” workflow, seamlessly transitioning from raw sample processing to analytical result output [262].

To address the limitations of traditional pesticide residue detection methods, such as insufficient detection sensitivity, high time and labor costs, inability to perform real-time monitoring, and susceptibility to interference in detection results [263], a study is proposed that combines microfluidic platforms with surface-enhanced Raman scattering (SERS) technology to achieve continuous, trace-level, and rapid detection (Figure 10B).

The core advantage of fully integrated microfluidic biochips lies in their exceptional degree of functional integration, which eliminates complex sample pretreatment requirements and enables direct field deployment [151,264]. These systems simultaneously deliver high sensitivity and specificity for trace-level contaminants, establishing a rapid, accurate, and field-portable methodology for agricultural safety screening [265].

#### 4.1.2. Live Cell Sensor

The core of living cell sensors based on genetically engineered bacteria for detecting antibiotic residues lies in utilizing the specific recognition and signal transduction mechanisms within bacteria [266]. Through genetic engineering modification, antibiotic-responsive elements are tandemly introduced into bacteria along with reporter genes [267]. When the target antibiotic is present in the environment, it binds to the specific receptor within the bacterium (Figure 10C), triggering a conformational change in the receptor, which in turn activates the expression of the downstream reporter gene. The expression products of the reporter gene can be detected by means of fluorescence intensity, color change, or enzyme activity, thereby indirectly reflecting the presence and concentration of the antibiotic [268].

#### 4.1.3. Molecularly Imprinted Polymer (MIP) Sensors

Molecularly imprinted polymer (MIP) sensors achieved unprecedented tolerance through innovations including a vinylimidazole-divinylbenzene copolymer framework and a hydrogen-bonding self-assembly strategy utilizing 4-vinylpyridine (4-VP) for high-fidelity molecular recognition of the aflatoxin B_1_ template [259]. These sensors demonstrated remarkable resilience: signal attenuation remained minimal after prolonged exposure to acidic citrus juice [269]; structural integrity was maintained at high temperatures in an oil phase, as verified by scanning electron microscopy (SEM) showing no collapse; and binding capacity retention was high after repeated cycles of hexane washing [270].

The analyzer features an MIP-detection probe that directly captures hydroperoxides within edible oils [271]. The instrument then precisely quantifies the peroxide value by measuring associated conductivity changes [269,272]. The exceptional tolerance of MIP sensors enables accurate pollutant detection within diverse and complex matrices (Figure 10D), significantly broadening their application scope [238,273].

### 4.2. Nanomaterial Technology

**Figure 11:**
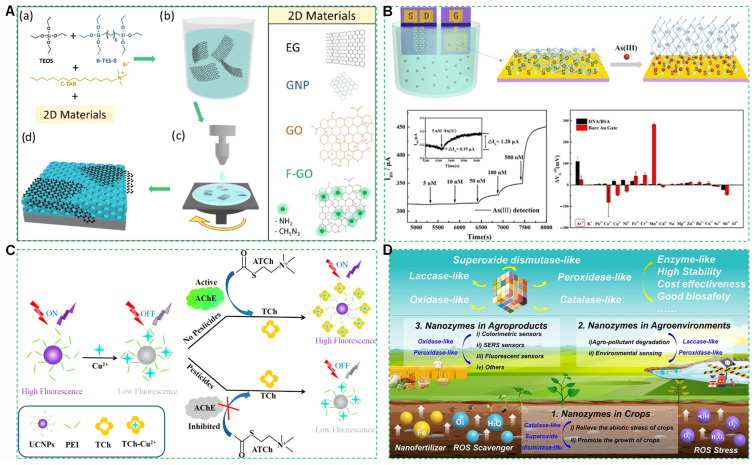
(**A**) Synthesis of the porous nanocomposite films: (**a**) Different types of 2D materials were directly added to the hybrid silica sol (**b**); (**c**) The films were prepared by spin-coating (**d**), which, after thermal treatment, allows the formation of a porous matrix embedding the graphene structures [299]. (**B**) DNA-gated graphene field-effect transistors for specific detection of arsenic (III) in rice [300]. (**C**) Schematic description of the *acetylcholinesterase* modulated UCNPs-Cu^2+^ fluorescence biosensor for organophosphorus pesticides [301]. (**D**) Classification of nanozymes currently applied in agriculture and their biocatalytic mechanisms [302].

Nanomaterial technology exhibits excellent performance in the detection of harmful components in agricultural products, and its core working principle stems from the unique physicochemical properties of nanomaterials [233,278]. Nanomaterials have a large specific surface area, with a very high proportion of surface atoms, and possess extremely strong surface activity and adsorption capacity, enabling them to interact specifically or non-specifically with harmful components in agricultural products [238,258]. The small size effect of nanomaterials allows them to easily enter microstructures and fully contact tiny harmful components in agricultural products (Figure 11A), thereby improving the sensitivity and accuracy of detection [279,280].

#### 4.2.1. Graphene Field-Effect Transistor (GFET) Sensors

The detection mechanism in GFET sensors involves the adsorption of organophosphorus pesticides onto the graphene surface. These adsorbed molecules act as electron acceptors, causing depletion of charge carriers (reduced carrier density) within the graphene channel [281]. A critical microfluidic sheath-flow configuration generates parallel laminar streams, effectively isolating the sample/sensor interface and eliminating most interference from particulate matter common in fruit or vegetable samples [282].

This GFET technology achieves an extremely low detection limit for chlorpyrifos and rapidly resolves methyl-parathion binding kinetics. A flexible GFET array deployed in situ on spinach leaves enables real-time pesticide residue tracking and can also be combined with DNA probe technology to detect heavy metal residues (Figure 11B). Unparalleled sensitivity and sub-second response kinetics enable continuous monitoring of trace agrochemical residues across the food supply chain, delivering a transformative platform for proactive food safety enforcement and quality assurance [283].

#### 4.2.2. Upconversion Nanoparticles (UCNPs)

The UCNPs achieve background suppression through excitation with a near-infrared laser, aided by their core–shell nanostructure [284]. This strategy effectively circumvents the intense autofluorescence interference common in cereal matrices within the ultraviolet-visible (UV-Vis) range [285,286,287], making it applicable for ultrasensitive detection of organophosphorus pesticides in food (Figure 11C). The large Stokes shift and enhanced quantum efficiency are critical attributes enabling this interference-free detection [288,289].

The aptamer-functionalized UCNP/graphene oxide (GO) Förster resonance energy transfer (FRET) platform exhibits significant fluorescence recovery upon aflatoxin B_1_ binding to the aptamer, yielding an ultrasensitive limit of detection well below China’s regulatory threshold [290,291]. Field deployment leverages a WHO-certified handheld scanner integrated with smartphone-based AI fluorescence analysis, enabling rapid toxin screening in grain silos and customs checkpoints [292,293]. Critically, UCNPs’ exceptional background suppression confers superior accuracy and reliability for quantifying toxins in complex grain matrices [294,295].

#### 4.2.3. Robust Nanozyme Stabilization Breakthrough

With the growing emphasis on sustainable agriculture, nanozymes, nanomaterials with enzyme-like activities but superior environmental durability and long-term stability compared to natural enzymes, endow agricultural technologies with enhanced performance, cost-effectiveness, and portability [296,297]. Benefiting from their multiple catalytic activities and renewable nano-characteristics, they integrate enzyme engineering and nanoscience to excel in agricultural scenarios, serving as a sustainable toolbox for improving agricultural production and reducing risks in agricultural systems (Figure 11D). Nanozymes leverage exposed Fe^2+^/Fe^3+^ catalytic sites on Fe_3_O_4_ nanoparticles to mimic natural peroxidase activity, driving radical chain reactions. Critically, their crystalline facets maintain structural integrity across pH 2.0–12.0, fully remediating the environmental sensitivity limitations inherent to natural enzymes [297,298].

This innovation achieves three revolutionary advances: (1) The zirconium dioxide (ZrO_2_) interlayer utilizes high-affinity coordination bonds to achieve near-irreversible metal ion confinement, greatly reducing ferrous ion release; (2) This design enhances ion immobilization efficiency by a huge margin compared to natural enzymes, resolving catalytic deactivation caused by ion leakage; (3) After high-temperature/long-hour accelerated aging, nanozymes retain high peroxidase activity, significantly outperforming natural horseradish peroxidase (HRP).

### 4.3. Genome Editing Technologies

#### 4.3.1. CRISPR-Cas Molecular Diagnostic System

**Figure 12:**
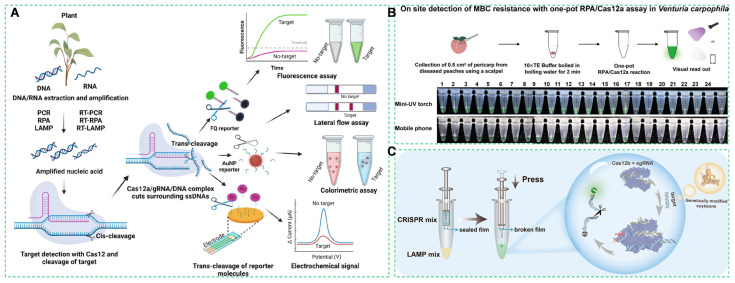
(**A**) Schematics of the Colateral Clease-Coupled CRISPR/Cas12a biosensing method [323]. (**B**) On site detection of MBC resistance with one-pot RPA/Cas12a assay in *Venturia carpophila* [324]. (**C**) A new method for detecting genetically modified soybeans [325].

The CRISPR/Cas system, composed of Cas endonucleases and guide RNAs [298], enables precise identification and cleavage of target nucleic acids [303]. Its inherent sensitivity, high specificity, and rapid assay time render it an effective alternative for diagnosing plant pathogens and identifying genetically modified crops (Figure 12A) [304].

The CRISPR-Cas12a/crRNA complex achieves single-molecule sensitivity through sequence-specific recognition of double-stranded DNA targets [305]. Upon target binding, the complex undergoes allosteric activation, unleashing collateral cleavage activity that degrades fluorophore-quencher-labeled single-stranded DNA (ssDNA) reporters [306]. This results in fluorescence signal amplification detectable at single-copy resolution, enabling ultrasensitive identification of exogenous genetic elements [307]. The CRISPR-Cas diagnostic platform achieves dual technological advancements: (1) Gold nanoparticle (AuNP)-enhanced signal amplification lowers the detection limit for the CP4-EPSPS transgene in *Zea mays*, far more sensitive than conventional PCR; (2) DNAzyme-mediated matrix pretreatment significantly reduces false-positive rates by specifically hydrolyzing starch polysaccharides in corn matrices [308]. This synergy establishes unprecedented sensitivity (attomolar-level detection) and exceptional anti-interference capability for on-site agricultural screening [309].

The CRISPR-Cas molecular diagnostic system delivers ultrasensitive detection, single-nucleotide specificity, and rapid field-compatible analysis [310]. This technology establishes a revolutionary methodology for monitoring genetically modified organisms (GMOs) and pathogenic microorganisms, achieving significantly enhanced sensitivity over PCR while greatly reducing false positives [311]. Beyond its diagnostic applications, the CRISPR/Cas genome-editing tool enables precise gene modification and holds enormous potential in crop improvement [312]. It can enhance plants’ tolerance to biotic and abiotic stresses, as well as improve yield and quality. Its diverse systems further support such precise modifications, contributing to sustainable crop improvement.

#### 4.3.2. RPA-CRISPR Cascade Amplification System 

The dual-engine cascade mechanism operates through two integrated phases: (1) It exponentially enriches target DNA under isothermal conditions, achieving substantial amplification rapidly [313]; (2) RPA amplicons activate Cas13a’s trans-cleavage activity, which degrades fluorogenic RNA reporters, generating significant fluorescence enhancement [314]. This integrated workflow delivers sample-to-answer detection in a short time, reducing turnaround time compared to standard RT-qPCR protocols and enabling field-deployable molecular diagnostics [315]. The RPA-CRISPR cascade amplification system enables on-site detection of benzimidazole resistance in *Venturia carpophila*, ensuring agricultural product safety (Figure 12B). It exhibits advantages including simplicity, rapidity, high sensitivity, high specificity, and ease of operation [316]. This system leverages isothermal amplification to eliminate dependence on thermal cycling equipment, enabling field-deployable diagnostics. As demonstrated in genetically modified (GM) soybean screening, the RPA-CRISPR cascade amplification achieves detection rapidly with an extremely low limit of detection (LOD), meeting stringent regulatory thresholds for GM component analysis. The assay’s rapidity and streamlined workflow significantly enhance accessibility for grassroots testing facilities and on-site monitoring applications [317].

#### 4.3.3. Genetically Modified (GM) Component Screening

The GM screening platform employs an “AND”-logic dual-gRNA system: one gRNA targets the universal transgenic element Cauliflower Mosaic Virus (CaMV) 35S promoter, while the second locks onto line-specific cassettes [318]. Positive identification requires simultaneous activation of both signals, eliminating false positives from endogenous genes and thereby ensuring high specificity [319].

This screening methodology significantly enhances the accuracy of genetically modified (GM) component detection by eliminating false-positive outcomes [320]. As validated in soybean assays, the system achieves robust discrimination between GM and non-GM varieties, maintaining detection fidelity even with trace endogenous gene interference [321]. This capability is particularly relevant given the widespread application of transgenic technology across multiple fields and the continuously increasing global cultivation area of genetically modified crops (Figure 12C). Such technological advances establish a robust framework for regulatory compliance and labeling of GM agricultural products, ultimately safeguarding consumers’ rights to informed decision-making [322].

### 4.4. Artificial Intelligence Technology

#### 4.4.1. Deep Learning-Driven Spectral Intelligence

**Figure 13:**
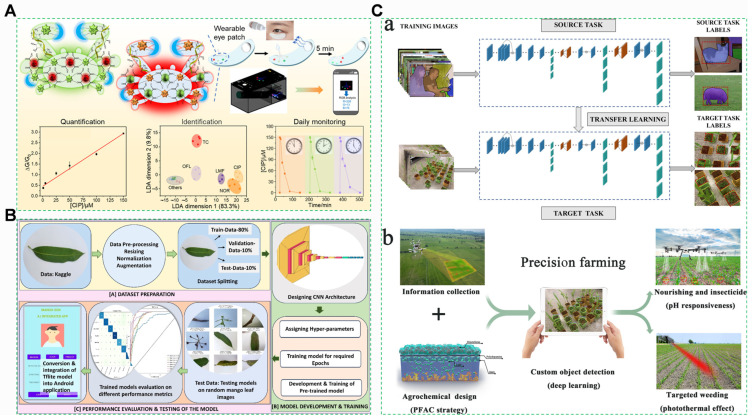
(**A**) A wearable, biocompatible, and dual-emission ocular multi-sensor patch for continuous analysis of fluoroquinolone antibiotics in tears [331]. (**B**) Workflow and process flowchart illustrating comprehensive workflow and process flowchart for developing, training, and implementing the CNN model for mango leaf disease classification [328]. (**C**) General architecture of deep learning for weed detection: (**a**) scheme of PFAC integrated with AI for weed management and intelligent nutrient and pesticide supply; (**b**) YOLO-v3, pretrained on COCO, completes training and testing on plant image datasets via transfer learning [332].

A Deep Residual Network (ResNet-152) architecture overcomes vanishing gradient limitations via cross-layer connections, enabling high-dimensional feature extraction from agricultural product spectra [326]. Progressive compression through multiple convolutional kernels reduces spectral fingerprint dimensionality to a small fraction of the original input, while a Softmax classifier simultaneously outputs both pesticide classification and concentration quantification [327]. Additionally, a deep learning model based on the residual network (ResNet-18), combined with Raman spectroscopy, enables rapid quantitative detection of multiple pesticides in *Malus pumila* and *Spinacia oleracea* (Figure 13A).

Convolutional Neural Networks (CNNs), as a milestone model in the field of deep learning, demonstrate their brilliance through the perfect integration of bionic design inspired by biological visual systems and engineering implementation [328]. With the development of technology, a convolutional neural network (CNN) model has been proposed, which achieves an impressive accuracy in identifying eight classes of mango leaf diseases (Figure 13B).

Notably, breakthrough performance has been achieved in apple cuticle multi-residue analysis, with the model delivering three landmark advancements: (1) High identification accuracy for multiple pesticides; (2) A breakthrough limit of detection (markedly improved over traditional PLS modeling); (3) Sustained quantification precision under variations in cuticular wax thickness, effectively eliminating complex matrix interference [329]. This deep learning-driven spectral intelligence establishes a novel analytical paradigm for rapid multi-residue screening of agricultural products and precise release of agrochemicals (Figure 13C), enhancing both accuracy and throughput of spectroscopic analysis [330].

#### 4.4.2. Blockchain-IoT Integrated Traceability System

The system deploys portable biosensors for heavy metal detection, with data encrypted via the Secure Hash Algorithm 3 (SHA-3) and timestamped onto the IOTA Tangle, a lightweight blockchain protocol [333]. Furthermore, a private consortium chain spanning farms, processing plants, and logistics partners employs Practical Byzantine Fault Tolerance (PBFT) consensus to ensure immutable ledger integrity. Smart contracts autonomously trigger product quarantine within 2 s upon heavy metal contamination threshold violations.

Empirical implementation within China’s Greater Bay Area vegetable supply network demonstrates that DNA barcoding (SHA-3 hashed) enables full-chain traceability for every production batch, significantly reducing contamination response time and effectively rebuilding consumer trust through verifiable provenance [334]. This blockchain-IoT integrated traceability system establishes end-to-end agricultural product monitoring from farm to retail, significantly enhancing supply chain transparency and strengthening food safety governance [335,336].

#### 4.4.3. Digital Twin-Driven Predictive Paradigm Shift for Agricultural Contamination Risks 

Pollution migration and transformation simulation engine integrates a discrete-mesh fluid dynamics framework with multiphysics coupling systems [337]. It quantifies Cd ion diffusion in soils through porosity and permeability coefficients, resolves nitrate crop enrichment kinetics via Michaelis-Menten equations, and dynamically calibrates these processes against real-time environmental parameters [338]. Key predictive breakthroughs include 72 h preemptive arsenic contamination alerts in rice paddies, increasing sampling efficiency; and precision modeling of chlorpyrifos vertical migration rates in sandy loam, establishing science-based pesticide application cycles [339]. This signifies a paradigm shift from empirical estimation to quantitative simulation in environmental risk assessment [340].

Real-time IoT sensors capture soil EC and foliar temperature data, dynamically assimilated via Extended Kalman Filter algorithms to iteratively refine pollutant diffusion models [341]. BeiDou high-precision positioning generates heavy metal heatmaps for targeted risk source localization [342]. In the same regional context, empirical implementation within China’s Greater Bay Area vegetable supply network demonstrates that complementary traceability technologies enable full-chain contamination monitoring, significantly reducing response time and effectively rebuilding consumer trust through verifiable data [343]. This transitions contamination control from reactive remediation to predictive governance [344]. Similarly, the main groups of emerging analytical technologies and their respective strengths and limitations are summarized in Table 3.

**Table 3:** Comparison of Emerging Detection Methods.

Technique Category	Typical Methods	Advantages	Disadvantages	SERS
Biosensors	Microfluidic chips, live-cell sensors, MIP sensors	High sensitivity; rapid on-site detection; potential for multiplexed analysis	Limited stability; relatively high cost; lack of standardized protocols	[345]
Nanomaterial-based Sensors	GFET, UCNPs, Nanozymes	Fast response; high sensitivity; strong anti-interference ability	Complex material synthesis; limited scalability for routine applications	[346]
Gene-based Detection	CRISPR-Cas, RPA-CRISPR	Ultra-high sensitivity and specificity; ideal for GMO and pathogen detection	Requires specific sample pretreatment; some systems rely on costly reagents	[347]
AI & Digital Technologies	AI-assisted spectral analysis, Blockchain traceability, Digital Twin systems	Powerful data processing; enables predictive monitoring and smart decision-making	Requires large datasets and computational resources; field application still in early stage	[348]

### 4.5. Summary of Strengths and Limitations of Emerging Methods

Emerging detection methods, including biosensors, nanomaterial-based platforms, CRISPR assays, and AI-driven digital technologies, have greatly expanded the toolbox for food safety monitoring. These techniques provide significant benefits: biosensors and microfluidic devices enable rapid and on-site analysis with high sensitivity; nanomaterial-assisted sensors enhance signal amplification and improve detection limits; CRISPR-based systems achieve remarkable specificity and ultra-low thresholds for nucleic acid detection; and digital technologies, such as AI-assisted spectral analysis and blockchain-based traceability, offer new opportunities for intelligent data interpretation and supply chain transparency.

Despite these advances, several challenges remain. Biosensors and nanomaterial-based platforms often face reproducibility and stability issues when applied to complex food matrices. CRISPR assays, although powerful, still require careful sample pretreatment and may involve high reagent costs. AI and other digital approaches depend on large, high-quality datasets and robust computational infrastructure, and their application in regulatory systems is still at an early stage.

Taken together, these strengths and limitations highlight the complementary role of emerging methods alongside conventional technologies. While they show strong potential for addressing the shortcomings of traditional approaches, further efforts are required to improve standardization, reduce costs, and validate their performance in large-scale food safety monitoring.

## 5. Challenges and Perspectives

### 5.1. Strategic Pathways for Technical Bottleneck Breakthroughs

Complex matrix interference remains a critical challenge in agricultural product detection. To address this, researchers have pioneered magnetic nanomaterial purification platforms. Carboxyl-functionalized magnetic multi-walled carbon nanotubes leverage π-π interactions to selectively sequester polyphenolic interferents in produce, thereby significantly enhancing target analyte recovery rates [349]. Additionally, customized catechin-targeted molecularly imprinted polymers (MIPs) effectively eliminate matrix effects under a wide range of extreme pH conditions, outperforming conventional C18 columns [350,351]. Furthermore, a dielectrophoretic separation unit coupled with zeta potential modulation clears milk fat micelles within a short time, enabling precise fluoroquinolone antibiotic detection in high-lipid matrices and resolving long-standing purification challenges in complex environments [352].

Future research must prioritize developing next-generation purification platforms with enhanced selectivity and throughput to achieve ultraprecise and reliable detection. Critical pathways include the synergistic integration of nanomaterials and molecular imprinting technologies to engineer advanced sorbents exhibiting unprecedented selectivity and adsorption capacity, which are essential for confronting increasingly complex and demanding matrices [353].

### 5.2. Breakthroughs in China’s Reference Material System for Agri-Food Safety

Chlorpyrifos-oxidized metabolite trichloropyridinol was synthesized via CYP450 enzyme bioreactors and certified by the EU Reference Materials (ERM) program [354]. Engineered pARG plasmid reference materials (RMs) encoding multiple critical resistance genes achieved extremely low uncertainty, now formalized in ISO standards for antimicrobial resistance (AMR) detection. Under China’s leadership, the ISO/TC34 Working Group is developing RMs for pesticide-heavy metal co-contaminants, while FAO’s Resistome Database uses blockchain to archive and share AMR data across nations [355].

The development of RM systems continues to face significant challenges: (1) A lack of certified RMs for emerging pollutants, impeding accurate monitoring and regulatory compliance; (2) Suboptimal homogeneity and stability in existing RMs, particularly for labile compounds like mycotoxins and antibiotic residues [356,357]. To address these challenges, key strategies include: (1) Strengthening international collaboration through initiatives such as the nanomaterials working group to accelerate joint RM development, enabling data interoperability across nations [358]; (2) Deploying CRISPR-engineered biosensors for real-time stability monitoring of protein-based RMs; (3) Developing AI-driven predictive models to forecast RM degradation pathways under extreme conditions [359,360].

### 5.3. Breakthroughs in Microfluidics-Mass Spectrometry Integration

The Chip-MS integration has achieved transformative advances through innovations in core interface technology and system-wide performance metrics, particularly via a high-density silicon microneedle array ion source [361]. A silicon-based microneedle array chip with high pore density enables direct nanoliter sample ionization, achieving high ionization efficiency, a significant enhancement over conventional electrospray ionization. This architecture eliminates dead-volume interference in droplet transfer, which is critical for single-cell proteomics where sample loss is substantial in traditional systems [362].

The technology demonstrates breakthrough capabilities in rapid multi-residue screening, achieving high throughput (a significant increase over conventional LC-MS/MS methods) [363]. This is enabled by parallel microfluidic processing and automated sample injection, which minimize inter-run delays [364]. Nanomaterials, which show potential in liquid biopsy exosome research, and rapidly detectable chip SFC-MS technology hold significant value in agricultural detection [365]. Specifically, nanomaterials can separate and enrich exosome-like vesicles or biomarkers containing pathogen information from crop sap and irrigation water [366]; when combined with chip SFC-MS, they enable rapid, highly sensitive analysis of trace pollutants and pest-related biomarkers in agricultural products, supporting agricultural quality and safety monitoring and pest early warning [367].

The evolution of Chip-MS integration will prioritize three transformative directions for agricultural applications: (1) Scaling to high sample throughput via parallelized microfluidic architectures and multiplexed ion injection interfaces [368]; (2) Achieving ultrahigh sensitivity through nano-electrospray ionization (nano-ESI) optimization and enhanced ion transmission efficiency; (3) Developing MEMS-based mass analyzers to lower unit cost, while integrating micro-vacuum pumps and ambient ionization sources for field-deployable systems [369]. Additional advancements include deploying deep learning-augmented platforms for real-time spectral interpretation (reducing false discovery rates in complex datasets) and coupling with spatial transcriptomics via edge-computing algorithms to resolve cellular heterogeneity at sub-micrometer resolution [370].

## 6. Conclusions

The evolution of detection technologies will pivot toward three interconnected frontiers: multi-residue synchronous analysis, field-deployable real-time monitoring, and non-targeted screening ecosystems.

First, multi-residue synchronous analysis is set to replace single-target assays through quantum dot-encoded multiplexed platforms capable of screening numerous pesticides and mycotoxins in a single run, while CRISPR microarray chips achieve parallel identification of transgenic exogenous genes via programmable gRNA hybridization. Complementing this, AI-enhanced high-resolution mass spectrometry (HRMS) non-targeted databases now cover most emerging contaminants—from PFAS metabolites to microplastic additives—through deep learning-driven spectral matching.

Second, field-deployable real-time monitoring disrupts laboratory-centric models: Smartphone-integrated hyperspectral modules enable on-site pesticide screening with ultra-low detection limits; lyophilized CRISPR-Cas12a reagents permit equipment-free pathogen detection with visual results rapidly; and IoT sensor networks drastically reduce contamination traceability latency via blockchain-validated data streams across distributed nodes.

Third, non-targeted screening ecosystems transcend targeted methods by integrating metabolomics-AI models to quantify pesticide dose–effect relationships on gut microbiota using convolutional neural networks (CNNs) trained on microbial metabolic profiles. Additionally, exposomics correlation establishes pollutant-health risk thresholds by linking biomarkers to inflammatory cytokines, while digital twin platforms simulate pollutant migration with an error margin below 5% via real-time assimilation of soil hydrology and climate data using physics-informed neural networks (PINNs).

Finally, through these technological innovations and paradigm shifts, the detection of harmful components in agricultural products will gradually transition from “passive monitoring” to “proactive prevention,” providing stronger technical safeguards for global food safety. Meanwhile, the establishment of a global standardization system and enhanced cross-regional cooperation will further drive the application and promotion of detection technologies, ultimately benefiting the health and well-being of all humanity.

## Figures and Tables

**Table 1:** Overview of Hazardous Components in Agricultural Products and Their Detection Methods.

Hazardous Component	Representative Examples	Traditional Methods	Emerging Methods	Strengths	Limitations	SERS
Pesticide Residues	Organophosphates, Neonicotinoids, Herbicides	HPLC, GC–MS/MS	Fluorescent probes, Biosensors (electrochemical, microfluidic)	High sensitivity; standardized protocols; real-time potential with biosensors	Chromatography is costly and slow; biosensors face stability and standardization issues	[162]
Heavy Metals	Cd, Pb, As	AAS, ICP-MS	LIBS, Graphene FET nanosensors	Accurate trace-level quantification; nanosensors allow portability	Lab-based methods are expensive; nanosensors not yet widely commercialized	[163]
Mycotoxins	Aflatoxin B_1_, DON, Fumonisin B_1_	LC–MS/MS, ELISA	SERS, Quantum dot immunoassays	High specificity; trace-level detection; rapid immunoassays	Antibody-based methods may cross-react; some need cold-chain storage	[164]
Microbial Contaminants	*E. coli* O157:H7, *Salmonella*, *Listeria monocytogenes*	Culture methods, PCR	CRISPR-Cas assays, Biosensors (fiber-optic, nanomaterials)	Molecular methods highly specific; rapid detection possible	Culture-based methods are slow; molecular assays still costly	[165]
Antibiotic Residues	Tetracyclines, Fluoroquinolones, Macrolides	HPLC, ELISA	Live-cell biosensors, Fluorescent probes	Sensitive detection; biosensors provide real-time monitoring	Traditional methods are time-intensive; biosensors require further validation	[149]
Genetically Modified Material	GMO maize, soybean	PCR, qPCR	CRISPR-Cas, Next-generation sequencing (NGS)	Genome-level specificity; high accuracy	Requires DNA extraction and specialized instruments	[166]

## Data Availability

No new data were created or analyzed in this study. Data sharing is not applicable to this article.
